# Signal Transduction of Improving Effects of Ibudilast on Methamphetamine Induced Cell Death

**DOI:** 10.31557/APJCP.2019.20.9.2763

**Published:** 2019

**Authors:** Reza Tahvilian, Komail Amini, Hossein Zhaleh

**Affiliations:** 1 *Pharmaceutical Sciences Research Center, School of Pharmacy, *; 3 *Substance Abuse Prevention Research Center, Health Institute, Kermanshah University of Medical Sciences, *; 2 *Department of Biology, Faculty of Sciences, Razi University, Kermanshah, Iran.*

**Keywords:** Methamphetamine, Ibudilast, cell death, PC_12_

## Abstract

**Objective:**

Interaction of methamphetamine and sigma (σ) receptors lead to up-regulation and activation of these receptors. The σ receptors induced apoptosis in some parts of the brain by increasing calcium, dopamine, ROS, mitochondrial pores and caspase activity. Ibudilast is a phosphodiesterase inhibitor and anti-inflammatory drug, which can decrease the inflammatory cytokines. Also, it has a neuroprotective effect. It seems that ibudilast can reduce the methamphetamine-induced cell death due to inhibition of σ receptors.

**Materials and Methods:**

There were seven treatments including; control: culture medium, Treatment 1: 1mM methamphetamine, Treatment 2: 1mM methamphetamine and 1nM ibudilast, Treatment 3: 1mM methamphetamine and 10nM ibudilast, Treatment 4: 1mM methamphetamine and 100nM ibudilast, Treatment 5: 1mM methamphetamine and 1uM ibudilast, Treatment 6: 1mM methamphetamine and 10uM ibudilast, and Treatment 7: 1mM methamphetamine and 100uM ibudilast. Finally, for inhibition of PKA, CREB, IP3 receptor, NMDA receptor, Sigma receptor antagonist, sigma receptor agonist, cells were preincubated with adding H89 dihydrochloride, 666-15, Heparin, Ketamine, BMY 14802, and Pentazocine. MTT and LDH tests were performed for cell viability and cytotoxicity measurement, respectively. In continuing, the caspase activity colorimetric assay kit used for caspase 3 activity diagnosis. Rhodamine-123 performed to detection of mitochondrial membrane potential. TUNEL test used to DNA fragmentation and apoptosis, Fura-2 used to Measurement of (Ca^2+^) ic and (Ca^2+^) m, and fluorescence microscope used to Measurement of antioxidant enzyme activities.

**Results:**

Ibudilast increased the cell viability and the rhodamine-123 absorbance in methamphetamin-treated PC1_2_ cells. It reduced cell cytotoxicity, caspase 3 activity, ic and m Ca^2+^ concentration, (OH) generation and DNA fragmentation in all concentrations of 1 nM t0 100 µM (p<0.05) by the optimal concentration of 100 µM, between our tested treatments.

**Conclusion:**

Ibudilast as a phosphodiesterase inhibitor can reduce the methamphetamine-induced cell death due to inhibition of σ receptors through cAMP production.

## Introduction

N-methyl-1-phenylpropane-2-amine so-called methamphetamine created in Japan, 1893 (Anglin et al., 2000; Sato, 2009) and at first was used to treatment of Attention-deficit hyperactivity disorder, idiopathic insomnia, and Narcolepsy. During World War II it was also used to relieve fatigue at low dosage (5-60 mg/day). Also, in western countries methamphetamine was subjected to abuse and it increased dosage (500-3, 500 mg/day) show psychotic and addictive features (Gillberg et al., 1997; Sato, 2008; Monti, 2015; Kotagal, 2017; Castells et al., 2018; Chigome et al., 2018). Methamphetamine abuses have increased in a few past decades on all of the word, notably in the USA. Methamphetamine have other common names such as Glass, crystal, ice, meth, and speed (Rasmussen, 2015; Ben-Yehuda and Siecke, 2018; Champion et al., 2018). Methamphetamine reduces dopamine reabsorption and acts as a chemical mediator in the brain neurons in long-term abuse (Abuse, 2006; Volkow et al., 2015; Graves et al., 2017). Long-time consumption of this drug leads to massive disruption in the midfrontal gray matter, right frontal white matter and right basal ganglia regions of the brain. Methamphetamine toxic effect on dopaminergic and serotonergic neurons is clear in rodent but not in humans. Methamphetamine abusers differences in the chemistry of the brain, low concentration of N-acetylaspartate and high concentration of choline-containing compounds and myo-inositol compared normal individuals (Ali, 2006). Abusers of methamphetamines show a lower level of dopamine D_2_ receptors, which leads to reduced metabolism in the orbitofrontal cortex and may cause a mechanism for compulsive drug intake and addicting process (Volkow et al., 2001).

Previous studies show decreased viability and increased apoptotic parameters in PC1_2_ cell line as rat adrenal medulla pheochromocytoma driven cells, after methamphetamine exposure (Pitaksalee et al., 2015; Wongprayoon and Govitrapong, 2017; Xiong et al., 2018). Shreds of evidence show that decreased dopaminergic neuron cells may result in signaling mechanisms of oxidative stress-mediated cell death (Kanthasamy et al., 2011). Previous studies suggested the mechanism of methamphetamine by activation of σ receptors. These receptors are involved in many effects of methamphetamine such as increased dopamine release, ROS, and apoptotic cell death (Ostenfeld et al., 2005; Schetz et al., 2007). cAMP plays a critical role in regulating the expression of σ receptors. Therefore, these receptors are considered suitable therapeutic targets (Nguyen et al., 2005).

Ibudilast (3-isobutyryl-2-isopropylpyrazolo[1,5-a]pyridine) is a phosphodiesterase inhibitor that involves in lymphocytes, endothelial cells, and glial cells also use to treatment of asthma, inflammatory and neurological diseases, including multiple sclerosis, neuropathic pain, and opioid addiction (Gibson et al., 2006; Rolan et al., 2009). Ibudilast introduced as a neuroprotective compound in rat nerve cell cultures not only by anti-inflammatory features but also by neurotrophic factor up-regulating effects (Mizuno et al., 2004). Regarding the opposite effects of ibudilast and methamphetamine on rat neural cell lines, we hypothesized that ibudlilast reduce the disruptive effects of methamphetamine on rat nerve cell cultures. Ibudilast was selected because it can produce the cAMP through the inhibition of phosphodiesterase. Increasing the production of the cAMP reduces the expression of the σ receptors, which can play a vital role in reducing the effects of methamphetamine.

Therefore we applied a range of ibudilast concentrations from 1 nM to 100µM against 1mM of methamphetamine and investigated viability by MTT test, cytotoxicity by LDH test, ROS production,caspase-3 activity, DNA fragmentation, Mitochondrial membrane potential, and Ca^2+^ concentration as apoptotic factors.

## Materials and Methods

*Cell Culture*

10% fetal bovine serum (FBS, Gibco), 1% non-essential amino acid (NEAA, Sigma), 2mM L-glutamine (Sigma), 100IU/ml penicillin (Sigma), and 100µg/ml streptomycin (Sigma) used as DMEM culture media (Gibco) supplement in T-25 cm^2^ tissue culture flasks for PC12 cells growth. The incubation of the cell cultures performed at 37ºC and under 5% CO_2_ Medium and this process was repeated every two days once. Trypsin-EDTA 0.25% (Sigma) used for cell culture trypsinization when the cells receive 70 to 80% of confluency. Finally, cells subcultured in 24-well cell culture plates at the density of 1×10^4^ cells/well.

*Cell Treatment*

A day after the cell plating, PBS, pH 7.4 used for cell washing. There were seven treatments including; control: culture medium, Treatment 1: 1mM methamphetamine, Treatment 2: 1mM methamphetamine and 1nM ibudilast, Treatment 3: 1mM methamphetamine and 10nM ibudilast, Treatment 4: 1mM methamphetamine and 100nM ibudilast, Treatment 5: 1mM methamphetamine and 1uM ibudilast, Treatment 6: 1mM methamphetamine and 10uM ibudilast, and Treatment 7: 1mM methamphetamine and 100uM ibudilast. In continuing, the cells placed in the incubator at 37◦C with 5% CO_2_. The cells were cultured in 0.2% BSA containing DMEM culture medium.

*Cell viability (%) measurement (MTT assay)*

MTT assay was used for cell viability quantification. The assay performed in the below manner: 200 μL of DMEM media containing 0.2% BSA added to 15×10^3 ^cells, which loaded into a 96-well plate. After 24h incubation, 200 μL of treatments media as described above, added to the wells. The cells separately incubated with different treatments medium for 24 hours.

*Cell Cytotoxicity measurement*

LDH Cytoxicity Detection Kit (Roche, Germany) was used for cytotoxicity measurement. Lactate dehydrogenase (LDH), which released from damaged or destroyed cells into the media, measured by this kit for cell cytotoxicity quantification. An increase of LDH activity in each treatment indicates that the treatment solution has cytotoxicity effects on cells, which leads to further cell death. 

*Caspase-3 Assay*

According to the caspase activity colorimetric assay kit (Bio-techne) manufacturer’s protocol and using a plate reader we measured the caspase-3 activity of lysates from treated cells. The experiments were repeated twice and data received from two independent experiments.

*Detection of mitochondrial membrane potential (MMP)*

For quantitative analysis, Rhodamine-123 as a cell-permeable cationic fluorescence probe, used for MMP measurement. 

*Quantification of apoptosis incidence*

All cells in this study fixed by 4% w/v paraformaldehyde in PBS, pH =7.4 for 10 min at room temperature. For identification of the apoptotic cells by TUNEL (Terminal Uridine deoxynucleotidyl transferase dUTP Nick End Labeling) staining, an in situ cell death detection kit (Roche) was used based on manufacturer’s protocol. 

*Measurement of (Ca* ^2+^ *) ic and (Ca* ^2+^ *) m*

For intracellular (Ca^2+^) ic measurements, PC12 cells were loaded with 4 μM fura-2 AM at 37 °C in a 5 % CO_2_ incubator for 20 min in a HCO3^- -^ buffered solution containing. Cells then washed twice and incubated in the HCO3^- -^ buffered solution for at least 20 min before use, while cells superfused at a constant perfusion rate of 2 ml/min with the HCO3^- - ^buffered solution equilibrated with 95% O_2_ and 5 % CO_2_ to maintain pH of 7.4. All experiments performed at 37 °C. The excitation wavelength altered between 340 and 380 nm, and the emission fluorescence recorded at 510 nm.

(Ca^2+^) ic values calculated using the equation described by Grynkiewicz (Grynkiewicz et al., 1985). Relative mitochondrial (Ca^2+^) m measured with the fluorescent probe Rhod 2-AM following methods described previously (Hoth et al., 1997).

*Measurement of antioxidant enzyme activities*

Briefly, in order to visualize intracellular ROS, cells were incubated with treatment media for 24 h, and then washed three times with Krebs–Ringer–Hepes (KRH) buffer, next cells incubated for 1 h at 37 °C. Fluorescence (Ex. 490 nm and Em. 525 nm) was visualized using a fluorescence microscope.

*Signaling and inhibitor treatment study*

For the study of signal transduction induced by Ibudilast on methamphetamine-treated cells, we have used some selective inhibitors for blocking the receptors and cell signaling enzymes in the cells. For this aim, one day after plating PC1_2_ cells, cells were washed with PBS. For inhibition of PKA, CREB, IP3, NMDA receptor, Sigma receptor (σR) antagonist, sigma receptor (σR) agonist, cells were preincubated with adding 200nM H89 dihydrochloride (Tocris), 100nM 666-15 (Tocris), 2µM Heparin (Sigma), 50µM Ketamine (Sigma), 5µM BMY 14802 (Sigma), 5µM Pentazocine (Sigma), alone for 30 minutes, respectively. Then, cells were cultured in treatment medium 1 (free serum culture medium supplemented with 1mM methamphetamine) or treatment medium 7 (free serum culture medium supplemented with 1mM methamphetamine and 100 µM ibudilast) for 24h. The cells were placed in the incubator at 37°C with 5% CO_2_ and cell viability, cell cytotoxicity, mitochondrial and intracellular Ca^2+^, ROS production, mitochondrial membrane potential, and caspase-3 activity were measured.

## Results

*Cell culture*

*Cell Viability (%)*

**Figure 1:**
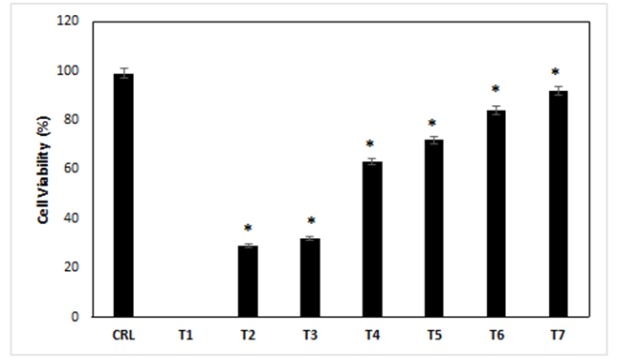
The Effects of Different Treatments on the Cell Viability of the Cells. CRL, culture medium; T1, 1mM methamphetamine; T2, 1mM methamphetamine and 1nM ibudilast; T3, 1mM methamphetamine and 10nM ibudilast; T4, 1mM methamphetamine and 100nM ibudilast; T5, 1mM methamphetamine and 1uM ibudilast; T6, 1mM methamphetamine and 10uM ibudilast; T7, 1mM methamphetamine and 100uM ibudilast. All data represented by mean ± S.E.M (p<0.05)

The cell viability was measured by MTT assay at 24h after the exposure of different concentrations of ibudilast and methamphetamine into the cells. The results of this experiment, in the case of control treatment, showed us 99% of cell viability. All the cells dead in the treatment-1, and the percentage of cell viability was 0%. The results showed that the viability of the cells in the 2-7 treatment media was decreased compared with control cells, respectively (p<0.05). Also, the cell viability of treatments 2-7 was increased compared with treatments 1 (p<0.05). The lowest and highest cell viability was for treatment 1 and treatment 7, respectively (Figure 1) (p<0.05).

*Cell cytotoxicity (%)*

**Figure 2:**
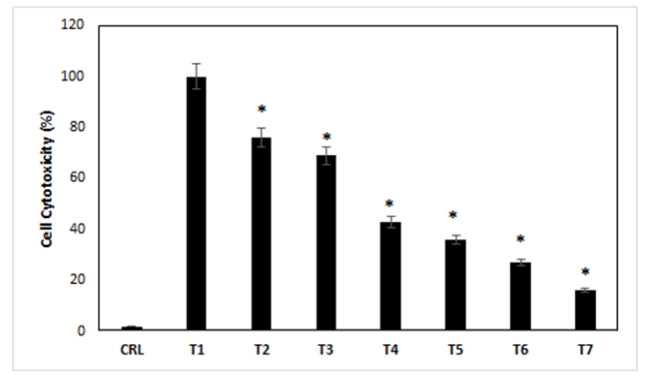
The Effects of Different Treatments on the Cell Cytotoxicity of the Cells. CRL, culture medium; T1, 1mM methamphetamine; T2, 1mM methamphetamine and 1nM ibudilast; T3, 1mM methamphetamine and 10nM ibudilast; T4, 1mM methamphetamine and 100nM ibudilast; T5, 1mM methamphetamine and 1uM ibudilast; T6, 1mM methamphetamine and 10uM ibudilast; T7, 1mM methamphetamine and 100uM ibudilast. All data represented by mean ± S.E.M (p<0.05)

24h after the exposure of different concentrations of ibudilast and methamphetamine into the cells, cell cytotoxicity measured by LDH assay. The results of this experiment, in the case of control treatment, showed us 2% of cell cytotoxicity. All cells dead in the treatment-1 and the percentage of cell cytotoxicity was 100%. The results showed that the cytotoxicity of the cells in the 2-7 treatment media increased compared with control cells (p<0.05). Also, the cell cytotoxicity decreased in treatments 2-7 compared with treatment-1, respectively (p<0.05). The highest and lowest cell cytotoxicity was for treatment-1 and treatment-7, respectively (Figure 2) (p<0.05).

*Cell death index*

**Figure 3:**
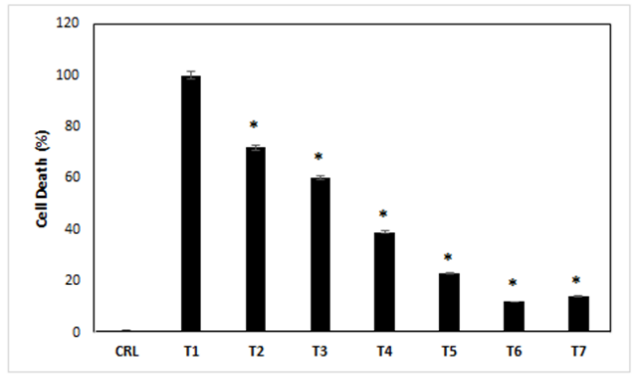
The Effects of Different Treatments on the Cell Death Index. CRL, culture medium; T1,1mM methamphetamine; T2, 1mM methamphetamine and 1nM ibudilast; T3, 1mM methamphetamine and 10nM ibudilast; T4, 1mM methamphetamine and 100nM ibudilast; T5, 1mM methamphetamine and 1uM ibudilast; T6, 1mM methamphetamine and 10uM ibudilast; T7, 1mM methamphetamine and 100uM ibudilast. All data represented by mean ± S.E.M (p<0.05)

The cell death index measured by TUNEL assay at 24h after the exposure of different concentrations of ibudilast and methamphetamine into the cells. The results of this experiment, in the case of control treatment, showed us 1% of cell death. All cells were dead in the treatment 1, and the percentage of cell death index was 100%. The results showed that cell deaths in the 2-7 treatment media were increased compared with control cells (p<0.05). Also, cell death decreased in treatments 2-7 compared with treatments 1, respectively (Figure 3) (p<0.05).

*Caspase-3 Assay*

**Figure 4:**
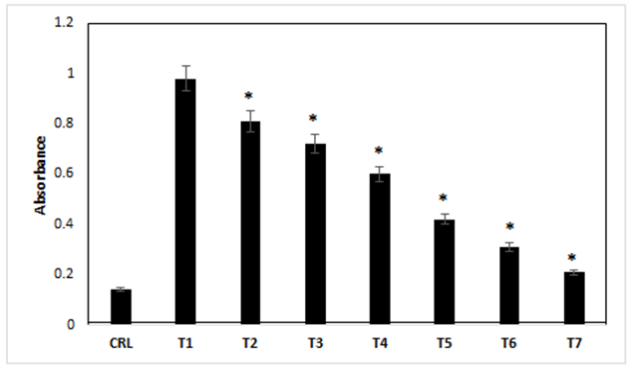
The Effects of Different Treatments on the Caspase-3 Activity of the Cells. CRL, culture medium; T1, 1mM methamphetamine; T2, 1mM methamphetamine and 1nM ibudilast; T3, 1mM methamphetamine and 10nM ibudilast; T4, 1mM methamphetamine and 100nM ibudilast; T5, 1mM methamphetamine and 1uM ibudilast; T6, 1mM methamphetamine and 10uM ibudilast; T7, 1mM methamphetamine and 100uM ibudilast. All data represented by mean ± S.E.M (p<0.05)

Caspase-3 activation is a sign of apoptosis, which eventually mediates by many caspases involved in this process. Furthermore, our results indicated that 2-7 treatments were increased in caspase-3 activity, after 24h, compared with control treatment (p<0.05). The lowest caspase-3 activation was in control cells, which was lower than other treatments (treatments 1-7) (p<0.05). Caspase-3 activation in treatments 2-7 was lower compared with treatment 1, respectively (Figure 4) (p<0.05).

*Mitochondrial membrane potential (Rhodamine-123 absorbance)*

Often, when apoptosis occurs through activating of caspase-3, a change in the mitochondrial membrane potential (Δφm) is visible. For more study in Δφm changing in the treated cells, cells exposed to different treatment media, then, Δφm measured by Rhodamine-123 staining and colorimetry assay at 24h after the exposure.

**Figure 5:**
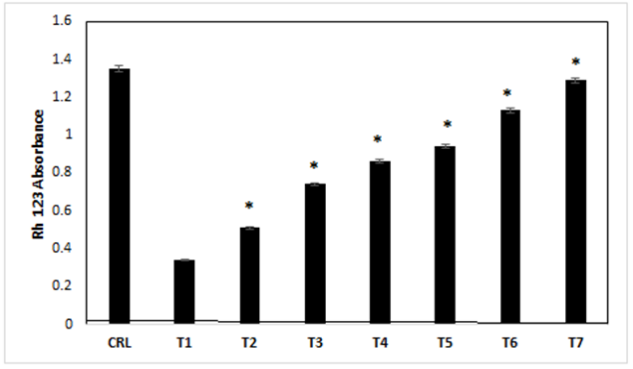
The Effects of Different Treatments on the Mitochondrial Membrane Potential (Rhodamine-123 Absorbance) of the Cells. CRL, culture medium; T1, 1mM methamphetamine; T2, 1mM methamphetamine and 1nM ibudilast; T3, 1mM methamphetamine and 10nM ibudilast; T4, 1mM methamphetamine and 100nM ibudilast; T5, 1mM methamphetamine and 1uM ibudilast; T6, 1mM methamphetamine and 10uM ibudilast; T7, 1mM methamphetamine and 100uM ibudilast. All data represented by mean ± S.E.M (p<0.05)

Based on our results, RH-123 absorption in all treatments after 24h decreased compared with control treatment (p<0.05). The RH-123 absorption in control cells was higher than other treatments (treatments 1-7) (p<0.05). RH-123 absorption in treatments 2-7 was higher compared with treatment 1, respectively (Figure 5) (p<0.05).

*(Ca* ^2+^ *) ic and (Ca* ^2+^ *) m*

**Figure 6:**
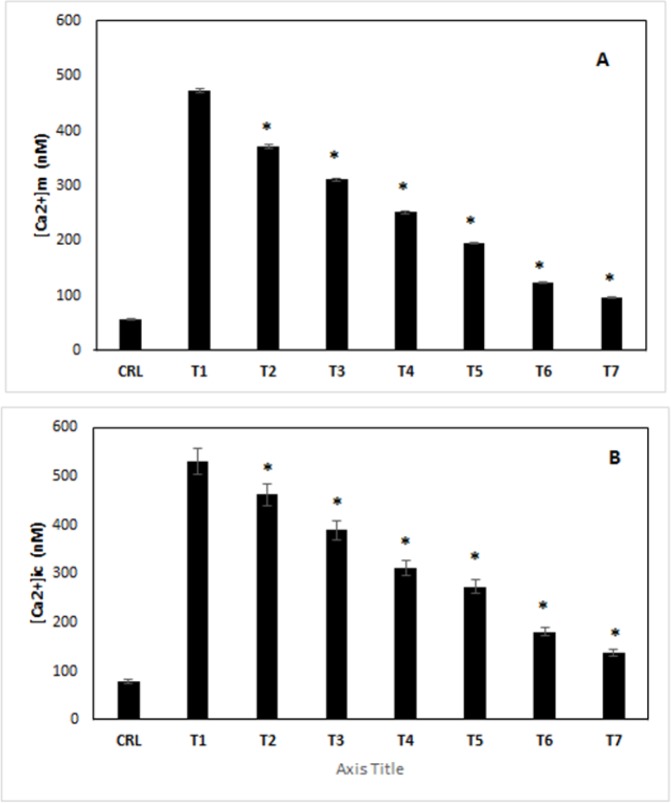
Determination of (Ca2+) m (A) and (Ca2+) ic (B) in Different Treated Cells. CRL, culture medium; T1, 1mM methamphetamine; T2, 1mM methamphetamine and 1nM ibudilast; T3, 1mM methamphetamine and 10nM ibudilast; T4, 1mM methamphetamine and 100nM ibudilast; T5, 1mM methamphetamine and 1uM ibudilast; T6, 1mM methamphetamine and 10uM ibudilast; T7, 1mM methamphetamine and 100uM ibudilast. All data represented by mean ± S.E.M (p<0.05)

Exposure of PC12 cells to different media had a special and clear effect on (Ca^2+^) ic and (Ca^2+^) m. In 2-7 treatments, the (Ca^2+^) ic were increased compared to control group (p<0.05) (Figure 6). Since ibudilast caused an early and sustained decrease in (Ca^2+^) ic in 2–7 treatments, we thought about that Ca^2+^ might have accumulated in mitochondria. In continuing, a microscope used to the assessment of changes in (Ca^2+^) m in cells loaded with the mitochondrial Ca^2+^ indicator. Following the treatment inhibitors comparison with treatment 1, we observed a significant decrease in (Figure 6) (Ca^2+^) m.

*Measurement of antioxidant enzyme activities*

**Figure 7:**
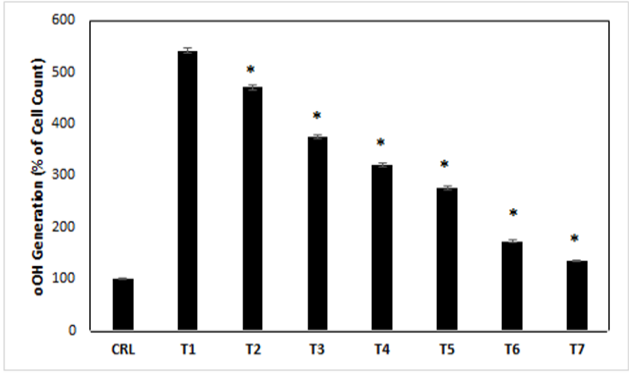
the antioxidants and reduce agents of endogenous reactive oxygen species (ROS) production in treated cells. CRL, culture medium; T1, 1mM methamphetamine; T2, 1mM methamphetamine and 1nM ibudilast; T3, 1mM methamphetamine and 10nM ibudilast; T4, 1mM methamphetamine and 100nM ibudilast; T5, 1mM methamphetamine and 1uM ibudilast; T6, 1mM methamphetamine and 10uM ibudilast; T7, 1mM methamphetamine and 100uM ibudilast. All data represented by mean ± S.E.M (p<0.05)

Exposure of PC12 cells to different treatment media had a clear effect on ROS (.OH) generation. Overload of intracellular and mitochondrial Ca^2+^ causes enhanced accumulation and Cytochrome C release in ROS pathway in treatment-1 cells. This event reverses in treatments 2-7 compared with treatment-1, respectively. In 2–7 treatments, the (.OH) generation increased compared with control cells (p<0.05). In 2–7 treatments, the (.OH) generation decreased compared with treatment-1 (Figure 7) (p<0.05).

*Signaling and inhibitor treatment study*

*Cell viability*

**Figure 8:**
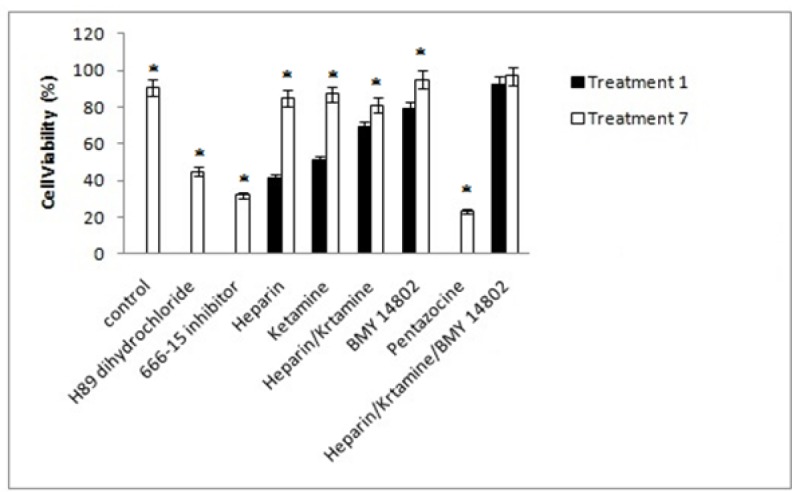
The Effects of Different Inhibitors on the Cell Viability of the Cells. Control, culture medium; Treatment 1, 1mM methamphetamine; Treatment 7, 1mM methamphetamine and 100uM ibudilast. All data represented by mean ± S.E.M (p<0.05)

The cell viability of treatments 1 and 7 was measured in the presence of inhibitors and activators of different signaling pathways. The inhibition of the PKA by H89 dihydrochloride and the inhibition of the CREB by the 666-15 inhibitor in the treatment1 resulted in whole cell death and the cell viability of the 0%. The cell viability of treatments 7 was higher than treatment 1 but lower than control. The inhibition of IP3 receptor by heparin and NMDA receptor by ketamine increased cell viability of the treatment 1. The cell viability of treatmen 7 was significantly higher than treatment 1. IP3 and NMDA receptors are regulators of calcium in ER and mitochondria, respectively. Simultaneous inhibition of IP3 and NMDA receptors also confirmed the effect of these calcium transporters on methamphetamine-induced cell death. The inhibition of σ receptor by BMY 14802 suppressed the amphetamine-induced cell death in treatments 1 and 7 but induction of σ receptor by Pentazocine significantly decreased the cell viability of treatments 1 and 7. The simultaneous inhibition of IP_3_, NMDA, and σ receptors showed that methamphetamine-induced cell death was suppressed in treatments 1 and 7. The results were summarized in Figure 8.

*Cell cytotoxicity*

The cell cytotoxicity of treatment 1 in the presence of PKA and CREB inhibitors was 100%. The cell cytotoxicity of treatment 7 was also significantly lower than treatment 1 and higher than the control group. The cell cytotoxicity of treatment 1 in the presence of IP3 and NMDA inhibitors was lower than control and higher than treatment 7 significantly. Simultaneous inhibition of the IP3 and NMDA receptors resulted in a significant decrease in methamphetamine-induced cell cytotoxicity. Inhibition of the σ receptor also reduced the cell cytotoxicity of methamphetamine in treatments 1 and 7, but the activation of this receptor did not have any effect on the reduction of methamphetamine-induced cell cytotoxicity. The simultaneous inhibition of IP3, NMDA, and σ receptors showed that methamphetamine-induced cell cytotoxicity was significantly decreased in treatments 1 and 7.

**Figure 9:**
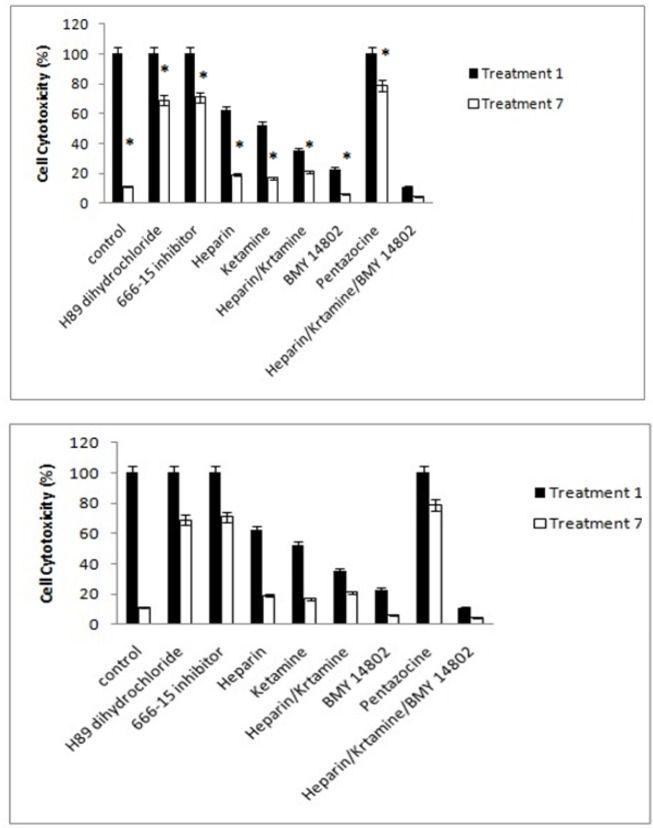
The Efects of Different Inhibitors on the Cell Cytotoxicity of the Cells. Control, culture medium; Treatment 1, 1mM methamphetamine; Treatment 7, 1mM methamphetamine and 100uM ibudilast. All data represented by mean ± S.E.M (p<0.05).

The results of the cell cytotoxicity of treatments 1 and 7 in the presence of inhibitors and activators of the main signaling pathways, which involve in methamphetamine function, are shown in Figure 9.

**Figure 10:**
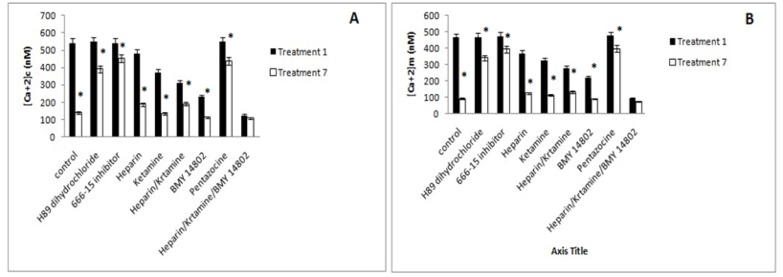
Determination of (Ca^2+^) c (A) and (Ca^2+^) m (B) in Different Inhibitor Treated Cells. Control, culture medium; Treatment 1, 1mM methamphetamine; Treatment 7, 1mM methamphetamine and 100uM ibudilast. All data represented by mean ± S.E.M (p<0.05)

**Figure 11:**
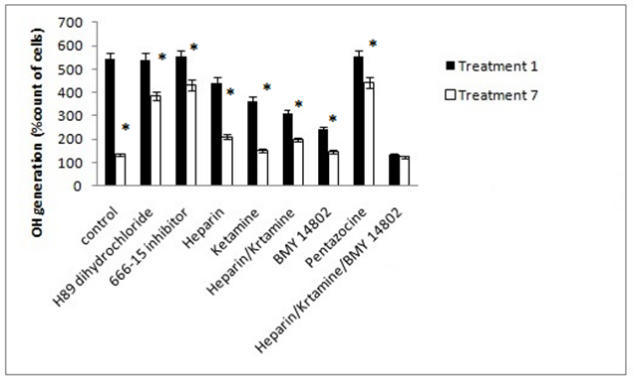
The Antioxidants and Reduce Agents of Endogenous Reactive Oxygen Species (ROS) Production in Inhibitor Treated Cells. Control, culture medium; Treatment 1, 1mM methamphetamine; Treatment 7, 1mM methamphetamine and 100uM ibudilast. All data represented by mean ± S.E.M (p<0.05)

**Figure 12:**
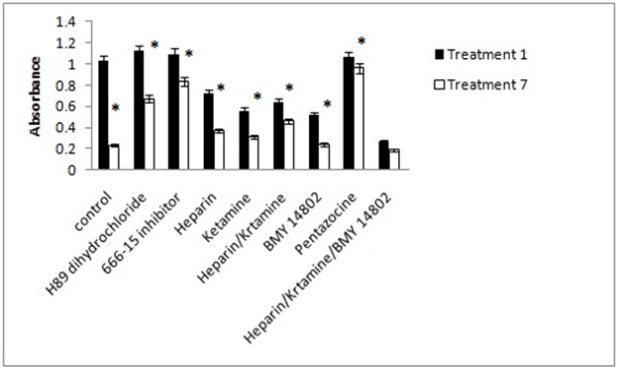
The Effects of Different Inhibitors on the Caspase-3 Activity of the Cells. Control, culture medium; Treatment 1, 1mM methamphetamine; Treatment 7, 1mM methamphetamine and 100uM ibudilast. All data represented by mean ± S.E.M (p<0.05)

*Mitochondrial and intracellular Ca* ^2+^

Inhibition of the PKA and CREB had no effect on the concentration of intracellular and mitochondrial Ca^2+ ^in treatment 1 and decreased the improvement effects of ibudilast in treatment 7. The inhibition of IP3, and NMDA receptors decreased intracellular and mitochondrial Ca^2+ ^concentrations in treatments 1 and 7 compared to the control. Concomitant inhibition of these Ca^2+^ transporters has a greater inhibitory effect on the intracellular and mitochondrial Ca^2+^ concentration increasing. The σ receptor inhibition also reduced the intracellular and mitochondrial Ca^2+^ concentration in treatments 1 and 7, but the activation of this receptor increased the concentration of intracellular and mitochondrial Ca^2+^ significantly. The simultaneous inhibition of IP3, NMDA, and σ receptors decreased the concentration of mitochondrial and intracellular Ca^2+^ concentration significantly. The results summarized in Figure 10.

*ROS production*

The study of ROS production was similar to that of intracellular and mitochondrial calcium concentrations. There was no significant difference in the ROS production of treatment 1 in the presence of PKA and CREB inhibitors. ROS production in treatment 7 was lower than treatment 1 and higher than control. The inhibition of IP3 and NMDA receptors individually reduced the ROS production in treatments 1 and 7, and this was more evident in the simultaneous use of two inhibitors. The σ receptor inhibition also reduced the ROS production in treatments 1 and 7, but the activation of this receptor increased the ROS production in these treatments. The simultaneous inhibition of IP3, NMDA, and σ receptors decreased the production of ROS significantly. The results shown in Figure 11.

*Caspase-3 activity*

The activity of caspase-3, which indicates the induction of apoptosis, was also investigated in our experiments. The inhibition of PKA and CREB does not reduced the activity of caspase-3 in treatment 1. Caspase-3 activity was decreased in treatment 7. It was higher than control and lower than treatment 1. The inhibition of IP3 and NMDA receptors individually reduced caspase-3 activity in treatments 1 and 7, and this was more evident in the simultaneous use of two inhibitors. The inhibition of the σ receptor reduced the activity of caspase-3 in treatments 1 and 7. The activity of caspase-3 in treatment 1 and 7 did not decrease in presence of σ receptor activator. The simultaneous inhibition of IP3, NMDA, and σ receptors decreased the caspase-3 activity of treatments 1 and 7 significantly. The results shown in Figure 12.

## Discussion

Ibudilast as a phosphodiesterase inhibitor increases the cAMP and activates the PKA pathway (Snider et al., 2012). In this study, we examined the neuroprotective role of ibudilast in the PC12 cell line. The neuroendocrine PC12 cell line, derived from rat pheochromocytoma as a tumor arising from chromaffin cells of the adrenal medulla. In our study ibudilast decreased cell cytotoxicity and increased cell viability which induced by methamphetamine. As the ibudilast concentration increased, toxicity decreased and viability increased. Ibudilast significantly suppressed neuronal cell death induced by methamphetamine.

Previous studies revealed that increase of σ receptors is a critical mechanism in methamphetamine-induced cell death via many events such as dopamine release, oxidative stress, ER stress, mitochondrial death cascade, excitotoxicity, and activation of microglia (Jayanthi et al., 2004; Riddle et al., 2006; Shen et al., 2008; Hall et al., 2009). The σ receptor, a group of drug binding sites, interacts with methamphetamine at a physiologically relevant concentration which lead to up-regulation and hyperactivation of σ receptors. σ1 and σ2 are two subtypes of σ receptors that increase the dopamine release which is one of initiating factors in neurotoxic effects of methamphetamine in the central nervous system (Matsumoto et al., 2003; Nguyen et al., 2005). The results of the study of the cell viability, cell cytotoxicity, Ca^2+^ concentration, ROS production, caspase-3 activity, and mitochondrial membrane potential in the presence of σ inhibitor showed that methamphetamine induced cell death by inhibiting this receptor and increasing calcium. Co-administration of σ, IP3, AND NMDA inhibitors showed that the methamphetamine-induced cell death was suppressed, and this confirmed that methamphetamine inactivates these signaling proteins in PC12 cells.

Dopamine causes stress on the ER through increased ROS production. Increasing the ER stress increases intracellular calcium and thus increases the mitochondrial pores, which ultimately induces cell death through mitochondrial pathway (Kaushal and Matsumoto, 2011). Studies have shown that the cAMP has a dual role in regulating σ receptors gene expression. cAMP down-regulates and up-regulates the σ receptor gene expression via PKA and ERK pathways respectively (Cormaci et al., 2007). Our results showed that methamphetamine induced cell death through inhibition of PKA and, as a result, inhibition of CREB as a downstream molecule which regulates the cAMP concentration. The σ1 receptor acts as a chaperone in the mitochondrial-associated ER membrane (MAM) and plays an important role in regulating calcium levels through inositol triphosphate (IP3). Activation of the σ^2^ receptor also leads to an increase in intracellular and mitochondrial calcium, which causes cell death through apoptosis (Vilner and Bowen, 2000; Hayashi and Su, 2007; Cassano et al., 2009). Methamphetamine increases the glutamate (Glu) concentration of the mammalian brain and N-methyl-D-aspartate (NMDA) and AMPA receptors activated by high levels of Glu can increase the intracellular Ca^2+^ concentration and subsequently reactive nitrogen species (RNS) formation (Hendrickson et al., 2006; Cadet et al., 2007; Simoes et al., 2007). Also, our results revealed that inhibition of IP3 and NMDA receptors which located in the ER and membrane, respectively, showed that methamphetamine causes calcium accumulation and cell death, and ibudilast in treatment 7 suppressed the calcium accumulation through inhibition of these receptors. Methamphetamine causes the neurotoxic damages through the increase of oxidative stress. For example, a study showed that increased oxidative stress induced by methamphetamine could activate the apoptotic pathway in human neuroblastoma SH-SY5Y cells (Wang et al., 2008). Methamphetamine-mediated ROS generation causes apoptosis in rat hippocampal neural progenitor cells (rhNPC), deregulating of dynamin-related protein 1 (Drp1), which leads to mitochondrial fragmentation (Tian et al., 2009). We aimed to the investigation of ibudilast protective effects on the death of neurons. In the following, we measured the influence of this drug in various concentrations on the generation of hydroxyl radical, intracellular and mitochondrial Ca^2+^, caspase activity and number of TUNEL-positive in the pc12 cell line treated by methamphetamine. The results show that as the concentration increases, radical hydroxyl production decreases, which suggests a reduction in DNA damage and thus reduces apoptosis in nerve cells. NO production activates inflammatory and apoptosis pathways which can be reduced by ibudilast.

Methamphetamine causes the production of ROS and inflammatory cytokines such as IL-1, IL6, IL10, and TNFα and by creating an inflammatory pathway activates the pathway of apoptosis and increases the cytotoxicity of the nerve cells (Vaudry et al., 2002). Methamphetamine-induced neurogenesis inhibition leads to incomplete disruption of the cell replacement and repair, resulting in neurodegenerative disease phenotypes, so more study will clarify the relationship between the effect of methamphetamine abuse on the regeneration of the brain and neurodegenerative diseases. Ibudilast, as a type-specific inhibitor of phosphodiesterase 4 and 8, can suppress activated microglia-induced neuronal cell death. It can suppress the production of nitric oxide (NO), reactive oxygen species, interleukin (IL)-1β, IL-6, and tumor necrosis fator (TNF)- α, and On the other hand, it can increase the expression of the inhibitory cytokine, IL-10 (Mizuno et al., 2004) . Also, ibudilast treatment leads to increased production of NGF, GDNF, and NT-4 (Yoneda et al., 2001). Therefore, it can play a neuroprotective role with anti-inflammatory properties and the induction of an increase in the expression of neurotrophic factors. A previous study revealed that IL-1 and IL6 reduce neurogenesis. Reducing these two factors has beneficial effects on cellular protection. On the other hand, reduction of TNF- α considered as an important factor in cellular survival (Mizuno et al., 1994; Kambayashi et al., 1995). Specific and non-specific inhibitors of phosphodiesterase reduce the level of nuclear factor NF-κB. On the other hand, by phosphorylation of protein kinase A (PKA), they increase the amount of CREB and consequently increase the cAMP (Parry and Mackman, 1997). Reduction of the NF-κB causes iNOS expression and decreases in pro-inflammatory cytokines production and rising CREB increase neuronal survival (Frey et al., 1993). In this study, the results of the TUNEL test revealed that DNA fragmentation which occurred by methamphetamine treatment, significantly reduced by ibudilast dose-dependently. Caspase 3 activity also significantly decreases with increasing concentration of ibudilast. These results are consistent with a previous study which states that ibudilast prevents H_2_O_2_ production, Caspase 3 activity, and nuclear condensation, leading to anti-apoptotic activity (Takuma et al., 2001) Other phosphodiesterase inhibitors, such as Pentoxifylline, as a non-specific inhibitor and Vesnarinone, which inhibit phosphodiesterase 3, also contribute to the increase of the cell viability and decrease of the cellular cytotoxicity. Pentoxifylline blocks the TNF-α release in microglia, however, it no effect on the accumulation of nitrites. On the other hand, Vesnarinone inhibits IL-1, IL-6, and TNF-α but no effect on IL-10 (Xie et al., 2002; Chen et al., 2003; Koyama et al., 2010; Hotta et al., 2013). In addition to the above effects, ibudilast also increases IL-10, which also increases IL-1, IL-6, and TNF-α (Stenvinkel et al., 2005) and it seems to have a stronger performance in protecting neurons against damage in neurodegenerative diseases. Also, it can significantly reduce intracellular and mitochondrial Ca^2+^, as it reduces the number of TUNEL-positive. The increase in cellular Ca^2+^ induced by methamphetamine results in the mitochondrial membrane disruption, and subsequently release of Cytochrome C as an apoptotic signal. Ibudilast by reduction of intracellular and mitochondrial Ca^2+^ prevents apoptosis. Rhodamine-123 is suitable for measuring the mitochondrial membrane potential, and the stability of this substance represents cell death through apoptosis, not necrosis (Darzynkiewicz et al., 1992). Finally, the measurement of rhodamine 123 in methamphetamine-infected PC12 cell line showed us a significant increase among increasing ibudilast concentration, so this confirms the previous results regarding the anti-inflammatory and anti-apoptotic effects of ibudilast. 

The ibudilast in the treatment 7 by inhibition of PKA and CREB, which increases the production of cAMP, as well as inhibition of NMDA and IP3 receptors, inhibits the methamphetamine-induced cell death. The σ receptor inhibition by BMY 14802 has more effect on the improvement of methamphetamine-induced cell death and stimulation of the σ by the Pentazocine induces cell death, so it means that methamphetamine is an activator of the σ receptor and ibudilast through inhibition of this receptor inhibits the primary signal of methamphetamine-induced cell death.

In conclusion, in this study our findings demonstrate that ibudilast, as a phosphodiesterase inhibitor, suppressed methamphetamine-induced cell death significantly. Because phosphodiesterase inhibition by ibudilast leads to the production of surplus cAMP, it is suggested that ibudilast inactivates the σ receptors and these inactivated receptors suppress the effects of methamphetamine such as calcium release, ROS production, mitochondrial pores, and caspase activity in some parts of the brain. In all our experiments, the optimal concentration was 100 µM. According to our findings, we introduce ibudilast as a candidate to attenuate the effects of methamphetamines abuse and reduce the effects of neurodegenerative diseases such as Alzheimer and Parkinson which show inflammatory properties in nerve cells.
